# Association of Single-Nucleotide Variants in the Human Leukocyte Antigen and Other Loci With Childhood Hodgkin Lymphoma

**DOI:** 10.1001/jamanetworkopen.2022.25647

**Published:** 2022-08-08

**Authors:** Cheng Chen, Nan Song, Qian Dong, Xiaojun Sun, Heather L. Mulder, John Easton, Jinghui Zhang, Yutaka Yasui, Smita Bhatia, Gregory T. Armstrong, Hui Wang, Kirsten K. Ness, Melissa M. Hudson, Leslie L. Robison, Zhaoming Wang

**Affiliations:** 1 School of Public Health, Shanghai Jiaotong University, Shanghai, China; 2 Department of Epidemiology and Cancer Control, St Jude Children’s Research Hospital, Memphis, Tennessee; 3 College of Pharmacy, Chungbuk National University, Cheongju, Korea; 4 Department of Structural Biology, St Jude Children’s Research Hospital, Memphis, Tennessee; 5 Department of Computational Biology, St Jude Children’s Research Hospital, Memphis, Tennessee; 6 University of Alabama at Birmingham; 7 Department of Oncology, St Jude Children’s Research Hospital, Memphis, Tennessee

## Abstract

**Importance:**

Studies focusing on genetic susceptibility of childhood Hodgkin lymphoma (HL) are limited.

**Objectives:**

To identify genetic variants associated with childhood-onset HL vs adult-onset HL.

**Design, Setting, and Participants:**

This genetic association study was performed with 3 cohorts: the St Jude Lifetime Cohort Study (SJLIFE), initiated in 2007 with ongoing follow-up, and the original and expansion cohorts of the Childhood Cancer Survivor Study (CCSS), initiated in the 1990s with ongoing follow-up. Results of these genome-wide association studies (GWASs) were combined via meta-analysis. Data were analyzed from June 2021 to June 2022.

**Main Outcomes and Measures:**

Childhood HL was the focused outcome. Single-nucleotide variant (SNV, formerly single-nucleotide polymorphism) array genotyping and imputation were conducted for the CCSS original cohort, and whole-genome sequencing was performed for the SJLIFE and CCSS expansion cohort.

**Results:**

A total of 1286 HL cases (mean diagnosis [SD] age, 14.6 [3.9] years), 6193 non-HL childhood cancer cases, and 369 noncancer controls, all of European ancestry, were included in the analysis. Using step-wise conditional logistic regression, the odds ratios (ORs) for each of the 3 independent SNVs identified in the human leukocyte antigen (HLA) locus were 1.80 (95% CI, 1.59-2.03; *P* = 2.14 × 10^−21^) for rs28383311, 1.53 (95% CI, 1.37-1.70; *P* = 2.05 × 10^−14^) for rs3129198, and 1.51 (95% CI, 1.35-1.69; *P* = 6.21 × 10^−13^) for rs3129890. Further HLA imputation revealed 9 alleles and 55 amino acid changes that potentially conferred HL susceptibility. In addition, 5 non-HLA loci were identified: (1) rs1432297 (OR, 1.29; 95% CI, 1.18-1.41; *P* = 2.5 × 10^−8^; *r*^2^ = 0.55; D′ = 0.75 with previously reported rs1432295, *REL*); (2) rs2757647 (OR, 1.30; 95% CI, 1.18-1.42; *P* = 3.5 × 10^−8^; *r*^2^ = 0.59; D′ = 0.83 with previously reported rs6928977, *AHI1*); (3) rs13279159 (OR, 1.33; 95% CI, 1.20-1.47; *P* = 1.7 × 10^−8^; *r*^2^ = 0.75; D′ = 1.00 with previously reported rs2019960, *PVT1*); (4) rs3824662 (OR, 1.52; 95% CI, 1.33-1.73; *P* = 3.9 × 10^−10^; *r*^2^ = 0.91; D′ = 1.00 with previously reported rs3781093, *GATA3*); and (5) rs117953624 (OR, 1.98; 95% CI, 1.56-2.51; *P* = 1.5 × 10^−8^; minor allele frequency, 0.02), a novel uncommon SNV mapped to *PDGFD*. Twelve of 18 previously reported genome-wide significant non-HLA SNVs (67%) were replicated with statistically significant results.

**Conclusions and Relevance:**

In this genetic association study, a predominantly common and potentially unique genetic etiology was found between childhood-onset and adulthood-onset HL.

## Key Points

**Question:**

What genetic variants or genes are associated with the risk of childhood Hodgkin lymphoma?

**Findings:**

In this genetic association study nested within 3 childhood cancer survivor cohorts, 3 independent single-nucleotide variants (SNVs, formerly single-nucleotide polymorphisms) in the human leukocyte antigen (HLA) locus and 5 loci in non-HLA regions were found to be significantly associated with risk of childhood-onset Hodgkin lymphoma. Two-thirds of previously reported Hodgkin lymphoma–associated non-HLA SNVs were replicated in the analysis.

**Meaning:**

These findings suggest predominantly common and potentially unique genetic etiology between childhood-onset and adulthood-onset Hodgkin lymphoma.

## Teaser

This genetic association study evaluates the association of single-nucleotide variants in the human leukocyte antigen and other loci with childhood-onset Hodgkin lymphoma.

## Introduction

Hodgkin lymphoma (HL) originates from B lymphocytes and is characterized by the presence of Reed-Sternberg cells and their variants.^1^ The remarkably high cure rate of 87% is attributable to advances in therapeutics featuring multiagent chemotherapy with or without radiotherapy.^2^ HL is most frequently diagnosed among people aged 20 to 34 years (30.7%); children and young adults (<20 years) account for 12.2% of patients with HL.^3^ Despite its low incidence in children younger than 5 years, HL is the most common cancer among adolescents and young adults aged 15 to 19 years, and it has a favorable 5-year survival, exceeding 95%. However, late effects of chemotherapy and radiotherapy are common after the cure of childhood-onset HL, resulting in accelerated aging^4^ and excess late mortality.^5,6,7^

HL is classified into histological subtypes based on cellular composition and architecture; the 2 major subtypes are classical HL (approximately 95% of cases) and nodular lymphocyte predominant HL (approximately 5% of cases).^8^ Increased familial risk and high concordance between monozygotic twins provide evidence of genetic susceptibility to HL.^9^ In addition to genetic risk factors, environmental risk factors, such as Epstein-Barr virus (EBV) infection, are also causally related to specific HL subtypes.^10^

The most significant genetic association for HL is mapped to the human leukocyte antigen (HLA) region. HLA genes serve as the fundamental platform for immune surveillance and responsiveness to internal and external antigens and are implicated in a number of human diseases.^11^ HLA class I molecules present antigens primarily to cytotoxic T-cells to kill target cells, and HLA class II molecules stimulate antibody production in response to specific antigens. Thus, HLA genes are crucial for the development of autoimmune diseases, infectious diseases,^12^ and cancers. HL was the first human disease associated with HLA genes,^13^ and subsequent studies revealed that HLA alleles along with specific single-nucleotide variants (SNVs, formerly single-nucleotide polymorphisms; eg, rs6903608) are significantly associated with HL risk.^14,15,16,17^ In addition to the strong association between HLA and HL, genetic variants in non-HLA regions have been reported, including rs4459895 (*LPP *[OMIM 600700])^18^ and rs1432295 (*REL *[OMIM 164910]),^19^ among others.

Although the genetic etiology of HL has been investigated, studies among patients with childhood-onset HL are lacking. Research using cancer survivors to investigate cancer etiology is subject to potential survival bias, but the very favorable survival for pediatric HL makes this a reasonable population for consideration of such investigation. In a recent study, we demonstrated that germline pathogenic variants in *BRCA2* are associated with risk of pediatric or adolescent non-HL.^20^ Here, we followed a similar paradigm to leverage genetic data of HL survivors to identify new genetic variants in HLA and non-HLA regions that are associated with the risk of childhood-onset HL.

## Methods

### Study Populations

Three large cohorts of survivors of childhood cancer were included in the current genetic association study.^21,22,23^ The St Jude Lifetime Cohort Study (SJLIFE) was approved by the St Jude institutional review board (IRB), and the Childhood Cancer Survivor Study (CCSS) was approved by the IRB of each participating center. This study is covered by the IRB approval for the 2 cohort studies. The SJLIFE is a retrospective cohort study initiated in 2007 with prospective clinical follow-up of 5-year survivors of childhood cancer treated at the St Jude Children’s Research Hospital. The CCSS is a retrospective cohort study of 5-year survivors of childhood cancer diagnosed and treated at 31 institutions in North America. The CCSS includes an original cohort diagnosed between 1970 and 1986 and an expansion cohort diagnosed between 1987 and 1999. In both studies, self-report or proxy-report questionnaires were used to assess demographic characteristics; clinical information was abstracted from medical records.

### Genotype Data

SNV-array genotyping was conducted using the HumanOmni5M-4 version 1 (Illumina) for 5739 childhood cancer survivors in the CCSS original cohort, followed by genome-wide imputation using the Human Reference Consortium Panel as the reference on Michigan imputation server (dbGaP accession: phs001327.v2.p1). Whole-genome sequencing (WGS) with 30 folds was performed on DNA derived from peripheral blood samples from 4853 SJLIFE participants, including 4402 childhood cancer survivors and 451 noncancer controls,^24,25^ and buccal/saliva samples from 2998 survivors in the CCSS expansion cohort.^20^ The entire collection of WGS data for the SJLIFE and CCSS expansion cohorts is accessible at the St Jude Cloud.^26^ All participants providing biological material for DNA analysis provided written informed consent.

### Quality Control

Of the 5739 survivors in the CCSS original cohort, 4671 remained after exclusion of 416 individuals of non-European ancestry whose admixture coefficient for 1000 Genomes CEU population was less than 80% and 652 who were also enrolled in SJLIFE.^4^ The final CCSS original cohort set consisted of 631 HL cases and 4040 non-HL cases (ie, survivors who had any childhood cancer diagnosis except HL) (eFigure 1 in the Supplement). Only variants (n = 8.4 million) with minor allele frequency (MAF) greater than 0.01, Hardy-Weinberg Equilibrium (HWE) *P* > 1 × 10^−6^, and having passed other filtering (ie, applying–keep-filtered = PASS setting in vcftools command) were retained for association analysis.

Among the 4402 SJLIFE survivors, 467 were diagnosed with HL. A total of 451 community controls with no history of childhood cancer were included and frequency matched to survivors by age, sex, and race. We subsequently excluded individuals (HL cases and controls) per the following criteria: (1) non-European ancestry with admixture coefficient for the 1000 Genomes CEU population less than 80% (86 case participants; 58 control participants); (2) incorrect or incomplete clinical characteristics (5 control participants); (3) individuals showing cryptic relatedness with another study participant (1 case participant; 19 control participants). Finally, a total of 380 HL cases and 369 controls remained for further analysis (eFigure 1 in the Supplement).

Of the 2998 survivors in the CCSS expansion cohort, 2428 were available for further analyses based on the following exclusions: (1) failure of the coverage and mapping quality control (50 individuals); (2) unexpected duplicates (12 individuals); (3) samples with excessive heterozygosity (97 individuals); and (4) individuals of non-European ancestry with admixture coefficient for 1000 Genomes CEU population less than 80% (411 individuals). Among the 2428 survivors, 275 were diagnosed with HL and considered cases; the 2153 survivors with other diagnoses (non-HL) were considered controls for comparison (eFigure 1 in the Supplement).

Additional quality control was performed to filter low-quality variants derived from WGS data, including the following criteria: (1) minimum genotype quality score of 20; (2) minimum depth of 5; (3) minimum mean depth of 10; (4) HWE *P* > 1 × 10^−6^; (5) maximum missing rate of 10% across all samples; (6) MAF greater than 0.01; and (7) exclusion of nonbiallelic loci. A total of 6.9 million SNVs and small insertions and deletions were in common with 8.4 million genotyped or imputed variants from the CCSS original cohort.

### Statistical Analysis

Genome-wide association study (GWAS) analyses were performed in each of the 3 cohorts (SJLIFE, CCSS original, and CCSS expansion). A logistic regression model was analyzed for associations between HL cancer status and allelic dose of each variant (0, 1, or 2), with adjustments for sex and the top principal components derived from genotypes. Fixed-effects meta-analysis was used to combine the summary statistics from the 3 GWAS analyses. Heterogeneity between data sets was assessed using *I^2^* as well as *P *for heterogeneity, calculated from the Cochran *Q* statistic. Variants with a meta-GWAS *P* < 5 × 10^−8^ were deemed genome-wide significant.

To refine associations between HL and HLA regions, step-wise conditional logistic regression was used to identify independent SNVs. First, the top (most significant) SNV from meta-GWAS was added to the list of covariates, and conditional association analysis was performed in each cohort. Then, results of the 3 conditional analyses were combined via meta-analysis to identify the next top SNV (independent secondary signal). This process was repeated until no SNV in the meta-analysis had a *P* < 5 × 10^−8^.

PLINK (version 1.90b) was used for association analysis.^27^ Other statistical analyses were performed with R version 3.6.1 (R Project for Statistical Computing), and 2-sided *P* ≤ .05 was considered statistically significant. Manhattan plots, regional SNV association results, and quantile-quantile plots were generated with LocusZoom.^28^ Pairwise linkage disequilibrium (LD) values were obtained by choosing individuals of European ancestry from the 1000 genomes reference panel using LDlink.^28^

Direct imputing summary association statistics of HLA variants (DISH)^19^ was used to infer statistical associations between HL risk and HLA alleles, amino acids, and SNVs based on all the variants cataloged in the Type 1 Diabetes Genetics Consortium reference panel comprising 5225 individuals of European ancestry. Biallelic SNVs (n = 28 455) with meta-GWAS summary statistics in the HLA region (Genome Reference Consortium Human Build 38, chr6:28510120-33480577) were fed into the program. Stringent quality control was applied to filter the imputed variants (MAF ≥0.005; imputation score *r*^2^ ≥ 0.5) after the DISH imputation.

## Results

### Meta-analysis of 3 GWAS Data Sets

**Figure 1.**
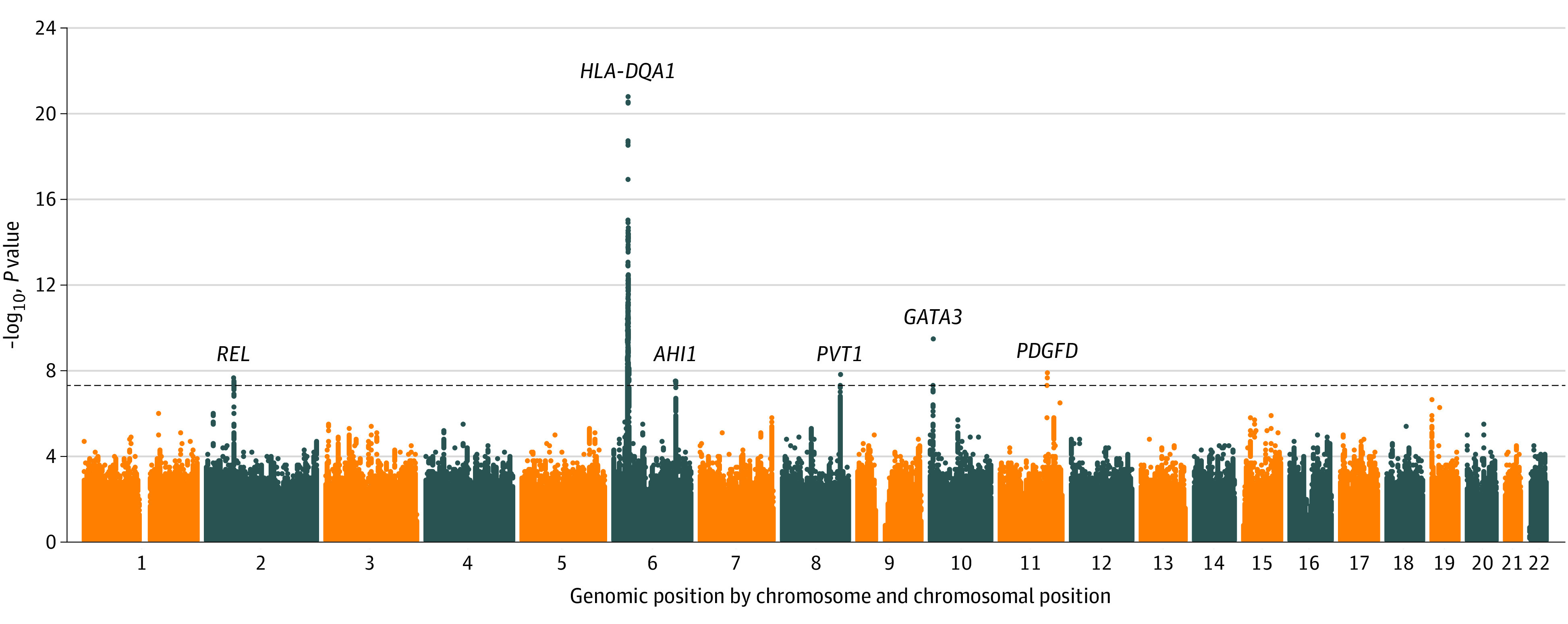
Manhattan Plot for Genome-Wide Associations With Hodgkin Lymphoma The overall landscape for meta-analysis of 3 genome-wide association studies are shown. The –log_10_ *P* values were plotted against the genomic position (x-axis), ie, chromosome and chromosomal position based on Genome Reference Consortium Human Build 38. The black horizontal dotted line represents the genome-wide significance threshold of *P* = 5 × 10^−8^. Genome-wide significant peaks were marked at 2p16.1 (*REL*), 6q21.3 (*HLA-DQA1*), 6q23.3 (*AHI1*), 8q24.21 (*PVT1*), 10p14 (*GATA3*), and 11q22.3 (PDGFD).

HL cases were diagnosed between 3.0 and 22.7 years of age (mean [SD] age, 14.6 [3.9] years). The summary statistics from the 3 GWASs were combined with a fixed-effects meta-analysis encompassing 1286 HL cases, 369 noncancer controls, and 6824 other childhood cancer cases, for a total of 6.88 million SNVs. We observed little evidence of genomic inflation by quantile-quantile plot of the meta-GWAS results (λ = 1.021) (eFigure 2 in the Supplement), suggesting little population stratification. The overall landscape of genome-wide associations and significant peaks are illustrated in Figure 1.

### SNVs in the HLA Region

**Figure 2.**
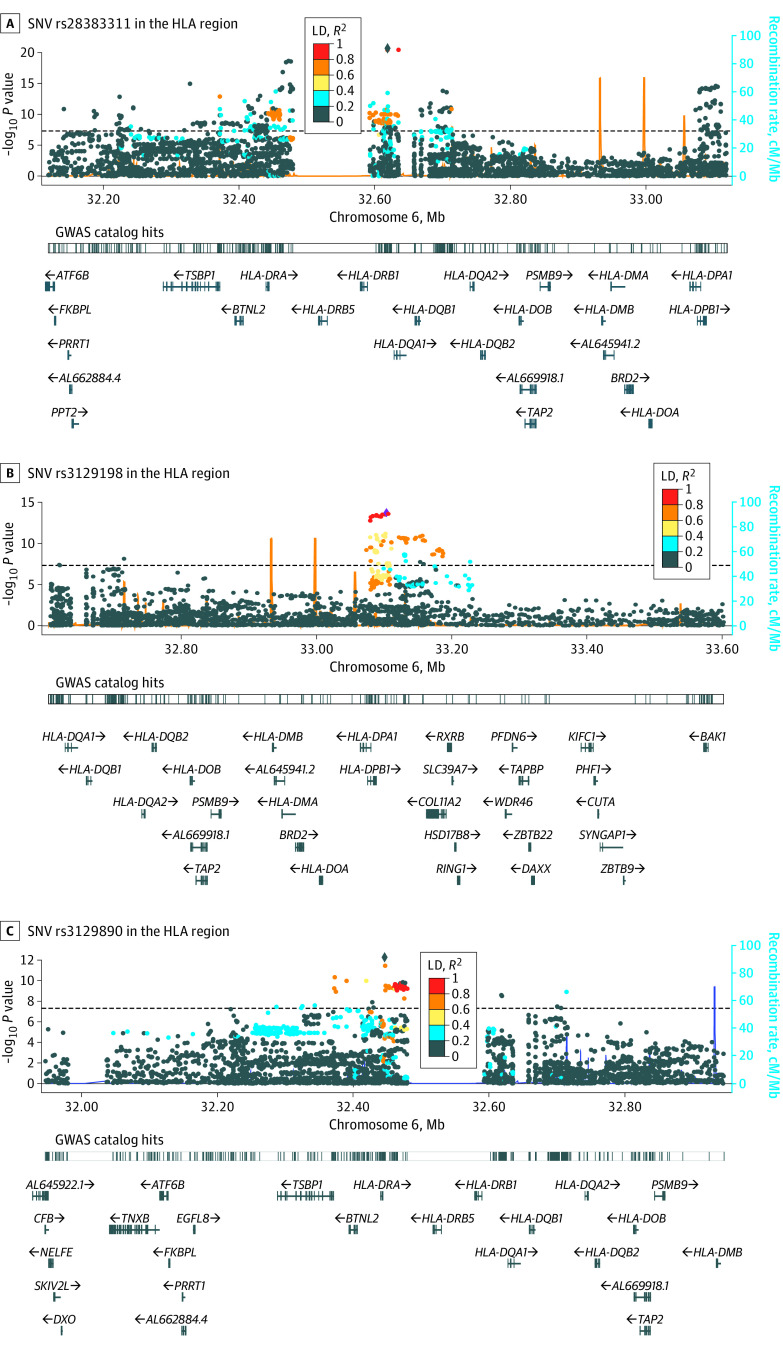
Regional Plot of Association Results and Recombination Rates for the Hodgkin Lymphoma Risk Loci The –log_10_ *P*-values (y-axis) are plotted against chromosomal position (x-axis). The black horizontal dotted line represents the genome-wide significance threshold *P* = 5 × 10^−8^. The most significant single-nucleotide variant (SNV) in each analysis is marked at the center of the 1-megabase (Mb) region nearby. The color intensity of each SNV represents the extent of linkage disequilibrium (LD) with the lead genetic variant, dark blue (*r*^2^ = 0) to red (*r*^2^ = 1.0). The light blue line at the bottom of each plot shows genetic recombination rates by using 1000 Genomes Project LD. The bottom tracks show nearby genes mapping to the region of association. Association hits, which have been reported in the EBI genome-wide association study (GWAS) catalog, are also annotated. Diamond-shaped markers indicate the lead SNV in each region, and circles indicate other SNVs in variable extent of LD with the lead SNV. Unconditional logistic regression results were plotted for A, and step-wise conditional logistic regression results were plotted for B and C.

A total of 691 SNVs in the HLA region were significantly associated with HL risk. The strongest association was marked by rs28383311 (OR, 1.80; 95% CI, 1.59-2.03; *P* = 2.14 × 10^−21^) (Table 1), in the neighborhood of *HLA*-*DRB1 *(OMIM 142857), *HLA*-*DQA1 *(OMIM 146880), and *HLA*-*DQB1* (OMIM 604305) genes (Figure 2A), which had a moderate LD (*r*^2^ = 0.46, D′ = 1.00) with previously reported SNV rs2858870 near *HLA*-*DRA* gene in adolescent and young adult-onset HL.^29^

**Table 1.** Association of Independent SNVs in the Human Leukocyte Antigen Locus With Hodgkin Lymphoma Susceptibility

SNV	Position^c^	Allele		Unconditional logistic regression		Conditional logistic regression^a^		Conditional logistic regression^b^	
		Risk	Other	OR (95% CI)	*P* value	OR (95% CI)	*P* value	OR (95% CI)	*P* value
rs28383311	32619234	A	T	1.80 (1.59-2.03)	2.14 × 10^−21^	NA	NA	NA	NA
rs3129198	33103045	A	G	1.54 (1.39-1.72)	2.64 × 10^−15^	1.53 (1.37-1.70)	2.05 × 10^−14^	NA	NA
rs3129890	32446496	T	C	1.23 (1.10-1.37)	1.71 × 10^−4^	1.48 (1.32-1.65)	7.04 × 10^−12^	1.51 (1.35-1.69)	6.21 × 10^−13^

Abbreviaitons: NA, not applicable; OR, odds ratio; SNV, single-nucleotide variant.

^a^ Conditional on rs28383311 and rs3129198.

^b^ Conditional on rs28383311.

^c^ Position according to human reference Genome Reference Consortium Human Build 38.

After conditioning on rs28383311, the secondary strongest association peak was marked by rs3129198 (OR, 1.53; 95% CI, 1.37-1.70; conditional *P* = 2.05 × 10^−14^) (Table 1), near *HLA-DPA1* (OMIM 142880) and *HLA-DPB1* (OMIM 142858) genes (Figure 2B). There was no LD (*r*^2^ = 0.00, D′ = 0.07) between rs3129198 and rs28383311 (eFigure 3 in the Supplement). However, rs3129198 had a perfect LD (*r*^2^ = 1.0, D′ = 1.00) with rs2281389 (*HLA-DPB1*) in a previous study comprising mostly adulthood-onset HL.^17^

In the next iteration of step-wise conditional logistic regression, we added rs3129198 and rs28383311 as covariates for adjustments in the model. A tertiary association signal emerged and was marked by the strongest associated SNV rs3129890 (OR = 1.51; 95% CI, 1.35-1.69; *P* = 6.21 × 10^−13^) (Table 1) in the neighborhood of *HLA-DRA* (OMIM 142860) (Figure 2C). The rs3129890 had a very low LD with the primary SNV rs28383311 (*r*^2^ = 0.06, D′ = 0.89) or the secondary SNV rs3129198 (*r*^2^ = 0.01, D′ = 0.12) (eFigure 3 in the Supplement) and a low LD with rs2395185 (*r*^2^ = 0.15, D′ = 1.00) near the *HLA-DRA* gene, which was reported previously by a study among HL cases diagnosed across a wide age range (16-80 years).^30^ Notably, rs28383311 and rs3129198 are mapped to HLA class II, whereas rs3129890 is mapped to the juncture between HLA class II and III. Previously reported HL-associated SNVs in the HLA region are provided in the eTable 1 in the Supplement. In addition, 10 of 14 previously reported genome-wide significant HLA SNVs (71%) were replicated with *P* < .05. Among the replicated SNVs, 8 of 10 (80%) were mapped to HLA class II; rs2248462 (*MICB* [OMIM 602436]) is mapped to the HLA I, and rs204999 (*PRRT1 *[OMIM 618297]) is mapped to the HLA III. Most (3 of 4 [75%]) of the remaining insignificant SNVs were mapped to the HLA class I region.

### Significant HLA Alleles and Amino Acid Changes

We identified 9 HLA alleles across 3 genes (6 for *DQB1 *[OMIM 604305], 2 for *DPB1 *[OMIM 142858], and one for *DRB1 *[OMIM 142857]) that had *P* values exceeding genome-wide significance (ie, <5 × 10^−8^); Seven alleles were positively associated with HL risk, and 2 alleles were negatively associated with it (Table 2). The HLA allele with the strongest association was HLA_DQB1_0603 (*P* = 9.35 × 10^−11^; imputed *Z* = 6.48). An additional set of 55 amino acid changes in HLA genes was significantly associated with HL risk (eTable 2 in the Supplement). Previously reported HLA alleles associated with HL susceptibility are provided in eTable 3 in the Supplement. All 6 HLA alleles in class II genes were significant, including HLA_DQB1_0602 and HLA_DRB1_1501, at the genome-wide significance level, whereas 2 HLA alleles in class I genes were not significant in our study. Regarding the 4 previously reported HL-associated amino acid changes, our analysis replicated the association for AA_DPB1_55_33156577_A (*P* = 3.6 × 10^−3^; imputed *Z* = 2.91) but not for the other 3 amino acid residues in HLA_DPB1 (ie, leucine 35, alanine 69, and valine 84).^31^

**Table 2.** Association of HLA Alleles With Hodgkin Lymphoma Susceptibility

Marker ID	Position^a^	Allele		Imputed Z^b^	*r*^2^pred^c^	Imputed *P* value
		Effect	Other			
HLA_DQB1_0603	32631061	Presence	Absence	6.48	0.82	9.35 × 10^−11^
HLA_DQB1_0602	32631061	Presence	Absence	6.08	0.88	1.17 × 10^−9^
HLA_DQB1_06	32631061	Presence	Absence	5.71	0.92	1.14 × 10^−8^
HLA_DQB1_0501	32631061	Presence	Absence	–5.54	0.97	3.07 × 10^−8^
HLA_DQB1_05	32631061	Presence	Absence	–5.87	0.92	4.41 × 10^−9^
HLA_DQB1_0301	32631061	Presence	Absence	5.81	0.88	6.08 × 10^−9^
HLA_DRB1_1501	32552064	Presence	Absence	5.84	0.89	5.28 × 10^−9^
HLA_DPB1_10	33049368	Presence	Absence	5.49	0.51	4.07 × 10^−8^
HLA_DPB1_1001	33049368	Presence	Absence	5.49	0.51	4.07 × 10^−8^

Abbreviation: HLA, human leukocyte antigen.

^a^ Postion refers to the genomic coordinates based on Genome Reference Consortium Human Build 37.

^b^ Z scores represent association with Hodgkin lymphoma.

^c^ The *r*^2^pred values represent the assessment of the imputation reliability at each HLA variant.

### SNVs in the Non-HLA Region

Our analysis identified the following genome-wide significant SNVs in non-HLA loci (Table 3). First, we identified rs1432297 (OR, 1.29; 95% CI, 1.18-1.41; *P* = 2.5 × 10^−8^) at 2p16.1, which is in moderate LD (*r*^2^ = 0.52, D′ = 0.73) with previously reported rs1432295,^19^ mapping to the *REL* gene. *REL* encodes c-Rel, which belongs to the Rel family of genes that regulate inflammation and immune response. REL is normally expressed in germinal B-cells and immunoblasts. Moreover, serving as a proto-oncogene, *REL* also functions in B-lymphocyte survival and proliferation^32^ and has been identified as a candidate gene for HL risk.^33^ Second, we identified rs2757647 (OR, 1.30; 95% CI, 1.18-1.42; *P* = 3.5 × 10^−8^) at 6q23.3, which is in moderate LD (*r*^2^ = 0.59, D′ = 0.83) with previously reported rs6928977,^18^ mapping to the *AHI1* (OMIM 608894) gene. *AHI1* is a prerequisite for development of the cerebellum and cortex in humans, and perturbations in *AHI1* expression may result in specific subtypes of human leukemia.^34^ Third, we identified rs13279159 (OR, 1.33; 95% CI, 1.20-1.47; *P* = 1.7 × 10^−8^) at 8q24.21, with a high LD (*r*^2^ = 0.75, D′ = 1.00) with previously reported rs2019960,^19^ located at a 82-Kb LD block telomeric to *PVT1 *(OMIM 165140), which represents a long noncoding RNA associated with acute myeloid leukemia and HL. Fourth, we identified rs3824662 (OR, 1.52; 95% CI, 1.33-1.73; *P* = 3.9 × 10^−10^) at 10p14, with a high LD (*r*^2^ = 0.91; D′ = 1.00) with previously reported rs3781093,^18^ mapping to an intronic region of *GATA3 *(OMIM 131320); *GATA3* expression is aberrant in Reed-Sternberg cells and their variants. In addition, a novel uncommon SNV with a large effect size, rs117953624 at 11q22.3 (OR, 1.98; 95% CI, 1.56-2.51; *P* = 1.5 × 10^−8^; MAF, 0.02), was identified and mapped to *PDGFD* (platelet-derived growth factor D; OMIM 609673) (eFigure 4 in the Supplement). Collective evidence supports a role for PDGF in HL pathogenesis,^35^ and PDGFD is a transforming, angiogenic growth factor.^10^ In addition, 12 of 18 previously reported genome-wide significant non-HLA SNVs (67%) were replicated with *P* < .05 (eTable 4 in the Supplement).

**Table 3.** Association of SNVs in Non–Human Leukocyte Antigen Regions With Hodgkin Lymphoma Susceptibility Based on Meta–Genome-Wide Association Studies

SNV	Position^a^	Neighboring genes	Allele		OR (95% CI)	*P* value
			Risk	Other		
rs1432297	chr2:60843517	*REL*	G	A	1.29 (1.18-1.41)	2.50 × 10^−08^
rs2757647	chr6:135446416	*AH1*	T	C	1.30 (1.18-1.42)	3.52 × 10^−08^
rs13279159	chr8:128150801	*PVT1*	G	A	1.33 (1.20-1.47)	1.74 × 10^−08^
rs3824662	chr10:8062245	*GATA3*	C	A	1.52 (1.33-1.73)	3.86 × 10^−10^
rs117953624	chr11:104438259	*PDGFD*	C	T	1.98 (1.56-2.51)	1.45 × 10^−08^

Abbreviations: OR, odds ratio; SNV, single-nucleotide variant.

^a^ Position according to human reference Genome Reference Consortium Human Build 38.

## Discussion

HL epidemiology is complex, with cases developing across an age spectrum, including children, adolescents, young adults, and older adults. Survival of HL is more favorable among children, adolescents, and young adults compared with older adults. Previous studies have suggested that a difference in HL etiology between children and adolescents vs older adults could explain this difference in survival.^36^ To investigate the genetic etiology of HL arising during childhood, adolescence, or young adulthood, we conducted a 3-way meta-GWAS analysis by leveraging preexisting data from the SJLIFE and CCSS.

Using step-wise conditional logistic regression methods, we identified 3 independent SNVs (rs28383311, rs3129198, rs3129890) in the HLA locus; most previous studies reported only 1 SNV.^17,19,29,30,37,38^ The primary and secondary SNVs were in moderate or high LD with previously reported SNVs, but the tertiary SNV had a low correlation with all established HLA SNVs, suggesting both the commonality and difference for genetic factors at the HLA locus underpinning HL risk during childhood and adolescence compared with other ages at onset.

We also identified 5 SNVs at non-HLA loci with genome-wide significance. Four of these SNVs were in moderate or high LD with previously reported SNVs, further validating these findings mapped to genes, such as *REL*, *AHI1, PVT1*, and *GATA3*^18,19,39^ in studies comprising mostly adult-onset HL. Collectively, these findings implicate multiple biological processes, including germinal center dysfunction, disrupted T-cell function, and nuclear factor–κB (NF-κB) activation, in HL pathogenesis^40^ across age groups. Our identification of rs117361561 (maps to *PDGFD*), a novel uncommon SNV with a large effect size, is intriguing because *PDGFD* has a fundamental role in cell growth, differentiation, survival regulation, invasion, and angiogenesis,^41^ supporting its potential association with cancer progression. Considering that NF-κB may moderate the expression of PDGF-inducible genes,^42^ the finding of a novel association between rs117361561 and HL risk provides additional evidence to support the role of NF-κB activation in HL pathogenesis, particularly in HL developing in younger people. Future mechanistic studies are needed to demonstrate how these genes function in HL carcinogenesis.

### Limitations

This study has limitations. First, we used case-case comparisons in 2 GWAS analyses and a case-control comparison in 1 GWAS analysis. Using other cases as a control set is not ideal and may result in either false-negative or false-positive results. However, we evaluated the heterogeneity across 3 GWASs for reporting genome-wide significant findings to minimize false-positive results. Second, the HL cases included in this study all had 5 or more years of survival; patients who died before 5-year survival were not included. Third, we did not have detailed histologic subtype information, so stratified analysis based on histologic subtype was not carried out. Fourth, EBV infection status was unavailable for all patients with HL in CCSS. Among 380 patients with HL in SJLIFE, 65 (17%) had EBV status, with 15 (23%) EBV positive and 50 (77%) EBV negative. Previous studies have identified low prevalence of EBV-positive HL in adolescent and young adults compared with children younger than 10 years or adults older than 80 years,^43^ as well as the different etiology between patients with EBV-negative and EBV-positive HL.^44^ This appears to be consistent with our current results, which showed that none of the 3 HLA SNVs we identified were in the HLA I class implicated in EBV-positive HL.^45,46^ Fifth, the study was conducted among individuals of European ancestry, so the findings may be not be generalizable to other racial and ethnic groups.

## Conclusions

In summary, we refined HL associations in the HLA locus by identifying 3 independent SNVs. In non-HLA regions, we found 1 novel SNV and replicated the majority of previous findings, including 4 SNVs exceeding genome-wide significance. These findings suggest mostly common, as well as potentially unique, genetic factors underpinning HL risk across age groups. Future replication and subsequent functional investigations are warranted to elucidate the genetic architecture and biological mechanisms of HL susceptibility.

## Notes

Supplement. **eFigure 1. **Study Population Flow Diagram **eFigure 2. **Quantile-Quantile Plot for the *P *Values in the –log_10_ Scale for the Discovery GWAS Meta-analysis **eFigure 3. **Pair-wise Linkage Disequilibrium Among 3 Independent SNPs in HLA Region **eTable 1. **Meta-GWAS Results for Previously Published HL-Associated SNPs in HLA Region **eTable 2. **Significant Amino Acid Changes Associated With HL in HLA Genes **eTable 3. **Previously Reported HLA Alleles Associated With HL Susceptibility **eTable 4. **Meta-GWAS Results for Previously Published HL-Associated SNPs in Non-HLA Region **eFigure 4.** SNV rs117953624 near *PDGFD*

## References

[1] Küppers R. The biology of Hodgkin’s lymphoma. Nat Rev Cancer. 2009;9(1):15-27. doi:10.1038/nrc2542 19078975

[2] Connors JM, Cozen W, Steidl C, . Hodgkin lymphoma. Nat Rev Dis Primers. 2020;6(1):61. doi:10.1038/s41572-020-0189-6 32703953

[3] Cancer Stat Facts. Hodgkin lymphoma. Accessed July 7, 2022. https://seer.cancer.gov/statfacts/html/hodg.html

[4] Qin N, Li Z, Song N, . Epigenetic age acceleration and chronic health conditions among adult survivors of childhood cancer. J Natl Cancer Inst. 2021;113(5):597-605. doi:10.1093/jnci/djaa147 32970815 PMC8096366

[5] Mertens AC, Yasui Y, Neglia JP, . Late mortality experience in five-year survivors of childhood and adolescent cancer: the Childhood Cancer Survivor Study. J Clin Oncol. 2001;19(13):3163-3172. doi:10.1200/JCO.2001.19.13.3163 11432882

[6] Castellino SM, Geiger AM, Mertens AC, . Morbidity and mortality in long-term survivors of Hodgkin lymphoma: a report from the Childhood Cancer Survivor Study. Blood. 2011;117(6):1806-1816. doi:10.1182/blood-2010-04-278796 21037086 PMC3056636

[7] Williams AM, Mandelblatt JS, Wang M, . Accelerated aging and mortality in long-term survivors of childhood cancer: a report from the St. Jude Lifetime Cohort (SJLIFE). J Clin Oncol. 2021;39(15_suppl):10045. doi:10.1200/JCO.2021.39.15_suppl.10045

[8] Campo E, Harris HL, Jaffe ES, Pileri SA, Thiele J. *WHO Classification of Tumours of the Haematopoietic and Lymphoid Tissues*. World Health Organization; 2008.

[9] Mack TM, Cozen W, Shibata DK, . Concordance for Hodgkin’s disease in identical twins suggesting genetic susceptibility to the young-adult form of the disease. N Engl J Med. 1995;332(7):413-418. doi:10.1056/NEJM199502163320701 7824015

[10] Jarrett RF, Krajewski AS, Angus B, . The Scotland and Newcastle epidemiological study of Hodgkin’s disease: impact of histopathological review and EBV status on incidence estimates. J Clin Pathol. 2003;56(11):811-816. doi:10.1136/jcp.56.11.811 14600123 PMC1770114

[11] Trowsdale J, Knight JC. Major histocompatibility complex genomics and human disease. Annu Rev Genomics Hum Genet. 2013;14:301-323. doi:10.1146/annurev-genom-091212-153455 23875801 PMC4426292

[12] Kulkarni S, Martin MP, Carrington M. The yin and yang of HLA and KIR in human disease. Semin Immunol. 2008;20(6):343-352. doi:10.1016/j.smim.2008.06.003 18635379 PMC3501819

[13] Amiel J. Study of the leukocyte phenotypes in Hodgkin’s disease. In Teraski PI, ed. *Histocompatibility Testing*. Munksgaard; 1967:79-81.

[14] Klitz W, Aldrich CL, Fildes N, Horning SJ, Begovich AB. Localization of predisposition to Hodgkin disease in the HLA class II region. Am J Hum Genet. 1994;54(3):497-505. 8116619 PMC1918115

[15] Oza AM, Tonks S, Lim J, Fleetwood MA, Lister TA, Bodmer JG. A clinical and epidemiological study of human leukocyte antigen-DPB alleles in Hodgkin’s disease. Cancer Res. 1994;54(19):5101-5105. 7923125

[16] Marshall WH, Barnard JM, Buehler SK, Crumley J, Larsen B. HLA in familial Hodgkin’s disease: results and a new hypothesis. Int J Cancer. 1977;19(4):450-455. doi:10.1002/ijc.2910190403 844915

[17] Moutsianas L, Enciso-Mora V, Ma YP, . Multiple Hodgkin lymphoma-associated loci within the HLA region at chromosome 6p21.3. Blood. 2011;118(3):670-674. doi:10.1182/blood-2011-03-339630 21596858

[18] Sud A, Thomsen H, Law PJ, ; PRACTICAL consortium. Genome-wide association study of classical Hodgkin lymphoma identifies key regulators of disease susceptibility. Nat Commun. 2017;8(1):1892. doi:10.1038/s41467-017-00320-1 29196614 PMC5711884

[19] Enciso-Mora V, Broderick P, Ma Y, . A genome-wide association study of Hodgkin’s lymphoma identifies new susceptibility loci at 2p16.1 (*REL*), 8q24.21 and 10p14 (*GATA3*). Nat Genet. 2010;42(12):1126-1130. doi:10.1038/ng.696 21037568 PMC4268499

[20] Wang Z, Wilson CL, Armstrong GT, . Association of germline *BRCA2* mutations with the risk of pediatric or adolescent non-Hodgkin lymphoma. JAMA Oncol. 2019;5(9):1362-1364. doi:10.1001/jamaoncol.2019.2203 31343663 PMC6659356

[21] Howell CR, Bjornard KL, Ness KK, . Cohort profile: The St. Jude Lifetime Cohort Study (SJLIFE) for paediatric cancer survivors. Int J Epidemiol. 2021;50(1):39-49. doi:10.1093/ije/dyaa203 33374007 PMC8453382

[22] Hudson MM, Ehrhardt MJ, Bhakta N, ; Jude Lifetime Cohort. Approach for classification and severity grading of long-term and late-onset health events among childhood cancer survivors in the St. Jude Lifetime Cohort. Cancer Epidemiol Biomarkers Prev. 2017;26(5):666-674. doi:10.1158/1055-9965.EPI-16-0812 28035022 PMC5413397

[23] Robison LL, Armstrong GT, Boice JD, . The Childhood Cancer Survivor Study: a National Cancer Institute-supported resource for outcome and intervention research. J Clin Oncol. 2009;27(14):2308-2318. doi:10.1200/JCO.2009.22.3339 19364948 PMC2677920

[24] Qin N, Wang Z, Liu Q, . Pathogenic germline mutations in DNA repair genes in combination with cancer treatment exposures and risk of subsequent neoplasms among long-term survivors of childhood cancer. J Clin Oncol. 2020;38(24):2728-2740. doi:10.1200/JCO.19.02760 32496904 PMC7430217

[25] Wang Z, Wilson CL, Easton J, . Genetic risk for subsequent neoplasms among long-term survivors of childhood cancer. J Clin Oncol. 2018;36(20):2078-2087. doi:10.1200/JCO.2018.77.8589 29847298 PMC6036620

[26] St Jude Children’s Research Hospital. St Jude Cloud. Accessed July 8, 2022. https://www.stjude.cloud/

[27] Purcell S, Neale B, Todd-Brown K, . PLINK: a tool set for whole-genome association and population-based linkage analyses. Am J Hum Genet. 2007;81(3):559-575. doi:10.1086/519795 17701901 PMC1950838

[28] Pruim RJ, Welch RP, Sanna S, . LocusZoom: regional visualization of genome-wide association scan results. Bioinformatics. 2010;26(18):2336-2337. doi:10.1093/bioinformatics/btq419 20634204 PMC2935401

[29] Cozen W, Li D, Best T, . A genome-wide meta-analysis of nodular sclerosing Hodgkin lymphoma identifies risk loci at 6p21.32. Blood. 2012;119(2):469-475. doi:10.1182/blood-2011-03-343921 22086417 PMC3257012

[30] Urayama KY, Jarrett RF, Hjalgrim H, . Genome-wide association study of classical Hodgkin lymphoma and Epstein-Barr virus status-defined subgroups. J Natl Cancer Inst. 2012;104(3):240-253. doi:10.1093/jnci/djr516 22286212 PMC3274508

[31] Taylor GM, Gokhale DA, Crowther D, . Further investigation of the role of HLA-*DPB1* in adult Hodgkin’s disease (HD) suggests an influence on susceptibility to different HD subtypes. Br J Cancer. 1999;80(9):1405-1411. doi:10.1038/sj.bjc.6690536 10424743 PMC2363076

[32] Houldsworth J, Mathew S, Rao PH, . *REL* proto-oncogene is frequently amplified in extranodal diffuse large cell lymphoma. Blood. 1996;87(1):25-29. doi:10.1182/blood.V87.1.25.25 8547649

[33] Barth TF, Martin-Subero JI, Joos S, . Gains of 2p involving the *REL* locus correlate with nuclear c-Rel protein accumulation in neoplastic cells of classical Hodgkin lymphoma. Blood. 2003;101(9):3681-3686. doi:10.1182/blood-2002-08-2577 12511414

[34] Jiang X, Zhao Y, Chan WY, . Deregulated expression in Ph+ human leukemias of *AHI-1*, a gene activated by insertional mutagenesis in mouse models of leukemia. Blood. 2004;103(10):3897-3904. doi:10.1182/blood-2003-11-4026 14751929

[35] Güler N, Yilmaz S, Ayaz S, . The platelet-derived growth factor level (PDGF) in Hodgkin’s disease and non-Hodgkin’s lymphoma and its relationship disease activation. Hematology. 2005;10(1):53-57. doi:10.1080/10245330400020405 16019446

[36] Hjalgrim H. On the aetiology of Hodgkin lymphoma. Dan Med J. 2012;59(7):B4485. 22759852

[37] Delahaye-Sourdeix M, Urayama KY, Gaborieau V, . A novel risk locus at 6p21.3 for Epstein-Barr virus-positive Hodgkin lymphoma. Cancer Epidemiol Biomarkers Prev. 2015;24(12):1838-1843. doi:10.1158/1055-9965.EPI-15-0534 26404960

[38] Frampton M, da Silva Filho MI, Broderick P, . Variation at 3p24.1 and 6q23.3 influences the risk of Hodgkin’s lymphoma. Nat Commun. 2013;4:2549. doi:10.1038/ncomms3549 24149102 PMC5053363

[39] Cozen W, Timofeeva MN, Li D, . A meta-analysis of Hodgkin lymphoma reveals 19p13.3 *TCF3* as a novel susceptibility locus. Nat Commun. 2014;5:3856. doi:10.1038/ncomms4856 24920014 PMC4055950

[40] Sud A, Thomsen H, Orlando G, ; PRACTICAL Consortium. Genome-wide association study implicates immune dysfunction in the development of Hodgkin lymphoma. Blood. 2018;132(19):2040-2052. doi:10.1182/blood-2018-06-855296 30194254 PMC6236462

[41] Wang Z, Ahmad A, Li Y, . Emerging roles of PDGF-D signaling pathway in tumor development and progression. Biochim Biophys Acta. 2010;1806(1):122-130. doi:10.1016/j.bbcan.2010.04.003 20434526 PMC2885511

[42] Olashaw NE, Kowalik TF, Huang ES, Pledger WJ. Induction of NF-kappa B-like activity by platelet-derived growth factor in mouse fibroblasts. Mol Biol Cell. 1992;3(10):1131-1139. doi:10.1091/mbc.3.10.1131 1421570 PMC275677

[43] Glaser SL, Lin RJ, Stewart SL, . Epstein-Barr virus-associated Hodgkin’s disease: epidemiologic characteristics in international data. Int J Cancer. 1997;70(4):375-382. doi:10.1002/(SICI)1097-0215(19970207)70:4<375::AID-IJC1>3.0.CO;2-T 9033642

[44] Shannon-Lowe C, Rickinson AB, Bell AI. Epstein-Barr virus-associated lymphomas. Philos Trans R Soc Lond B Biol Sci. 2017;372(1732):20160271. doi:10.1098/rstb.2016.0271 28893938 PMC5597738

[45] Diepstra A, Niens M, Vellenga E, . Association with HLA class I in Epstein-Barr-virus-positive and with HLA class III in Epstein-Barr-virus-negative Hodgkin’s lymphoma. Lancet. 2005;365(9478):2216-2224. doi:10.1016/S0140-6736(05)66780-3 15978930

[46] Niens M, van den Berg A, Diepstra A, . The human leukocyte antigen class I region is associated with EBV-positive Hodgkin’s lymphoma: HLA-A and HLA complex group 9 are putative candidate genes. Cancer Epidemiol Biomarkers Prev. 2006;15(11):2280-2284. doi:10.1158/1055-9965.EPI-06-0476 17119058

