# Supplementary material for: Association of Single-Nucleotide Variants in the Human Leukocyte Antigen and Other Loci With Childhood Hodgkin Lymphoma
Source: JAMA Netw Open. 2022 Aug 8;5(8):e2225647. doi: 10.1001/jamanetworkopen.2022.25647 (PMC9361085; doi:10.1001/jamanetworkopen.2022.25647)
Supplement: Supplement. — eFigure 1. Study Population Flow Diagram eFigure 2. Quantile-Quantile Plot for the P Values in the –log10 Scale for the Discovery GWAS Meta-analysis eFigure 3. Pair-wise Linkage Disequilibrium Among 3 Independent SNPs in HLA Region eTable 1. Meta-GWAS Results for Previously Published HL-Associated SNPs in HLA Region eTable 2. Significant Amino Acid Changes Associated With HL in HLA Genes eTable 3. Previously Reported HLA Alleles Associated With HL Susceptibility eTable 4. Meta-GWAS Results for Previously Published HL-Associated SNPs in Non-HLA Region eFigure 4. SNV rs117953624 near PDGFD [file jamanetwopen-e2225647-s001.pdf]

Supplemental Online Content

Chen C, Song N, Dong Q, et al. Association of single-nucleotide variants in the human leukocyte antigen and other loci with childhood Hodgkin lymphoma. *JAMA Netw Open.* 2022;5(8):e2225647. doi:10.1001/jamanetworkopen.2022.25647

eFigure 1. Study Population Flow Diagram

eFigure 2. Quantile-Quantile Plot for the *P* Values in the $-\log_{10}$ Scale for the Discovery GWAS Meta-analysis

eFigure 3. Pair-wise Linkage Disequilibrium Among 3 Independent SNPs in HLA Region

eTable 1. Meta-GWAS Results for Previously Published HL-Associated SNPs in HLA Region

eTable 2. Significant Amino Acid Changes Associated With HL in HLA Genes

eTable 3. Previously Reported HLA Alleles Associated With HL Susceptibility

eTable 4. Meta-GWAS Results for Previously Published HL-Associated SNPs in Non-HLA Region

eFigure 4. SNV rs117953624 near *PDGFD*

This supplemental material has been provided by the authors to give readers additional information about their work.

© 2022 Chen C et al. *JAMA Network Open.*

eFigure 1. Study Population Flow Diagram

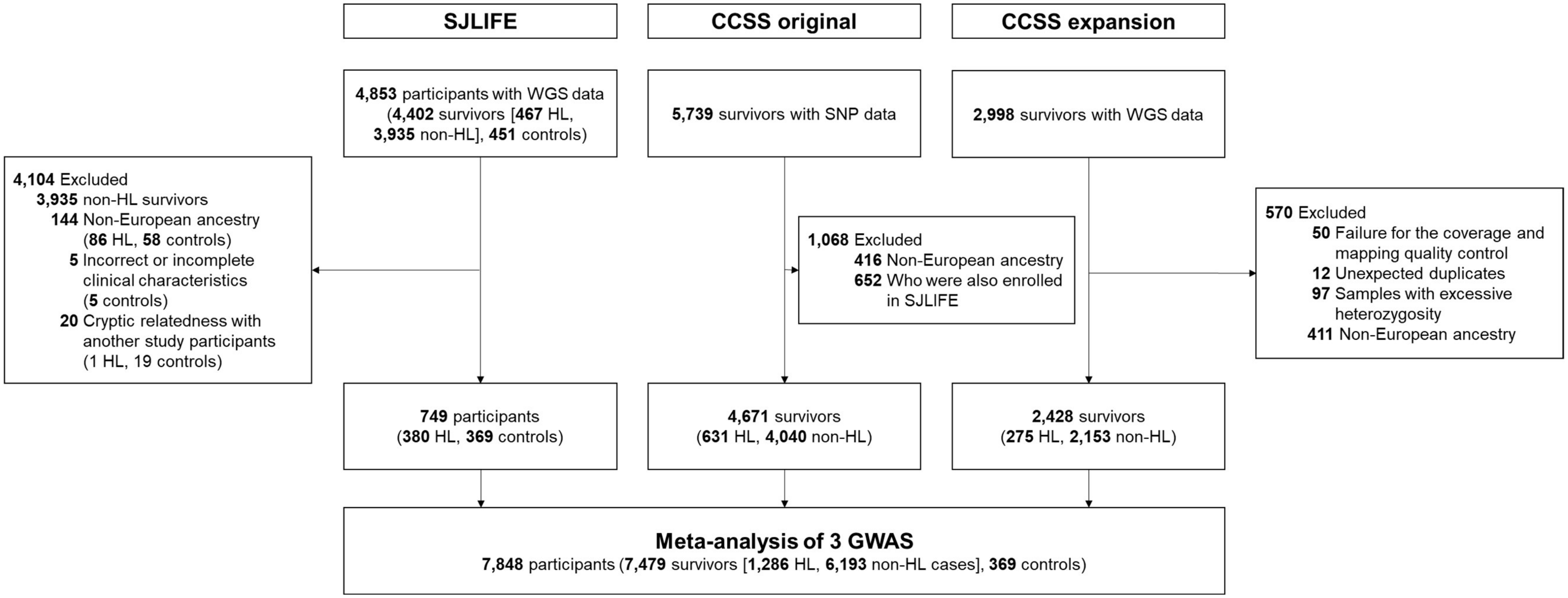

Abbreviations: SJLIFEE, St. Jude Lifetime Cohort Study; WGS, whole-genome sequencing; CCSS, Childhood Cancer Survivor Study; HL, Hodgkin lymphoma; SNP, single-nucleotide polymorphism; GWAS, genome-wide association study.

© 2022 Chen C et al. *JAMA Network Open.*

eFigure 2. Quantile-Quantile Plot for the *P* Values in the –$\log_{10}$ Scale for the Discovery GWAS Meta-analysis

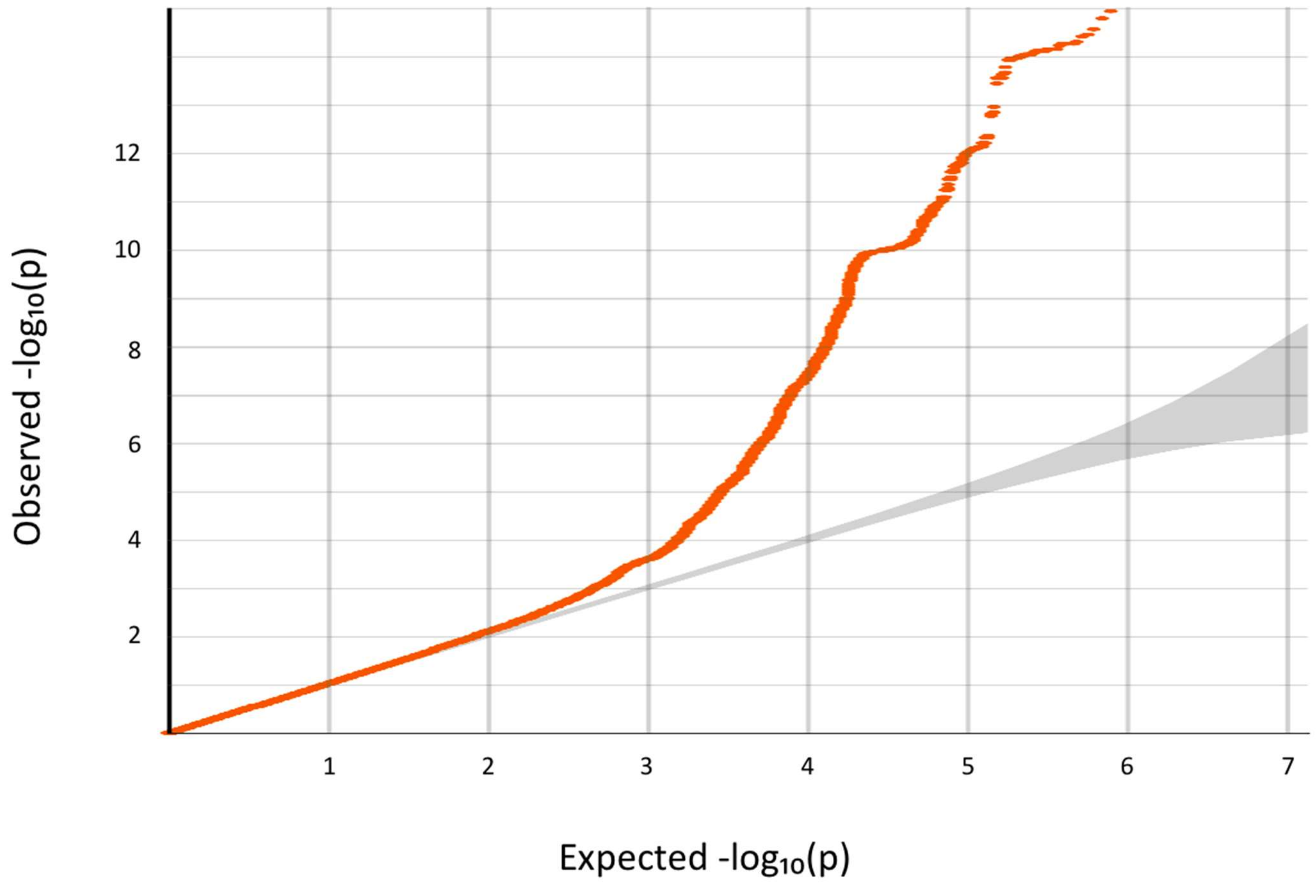

© 2022 Chen C et al. *JAMA Network Open.*

eFigure 3. Pair-wise Linkage Disequilibrium Among 3 Independent SNPs in HLA Region

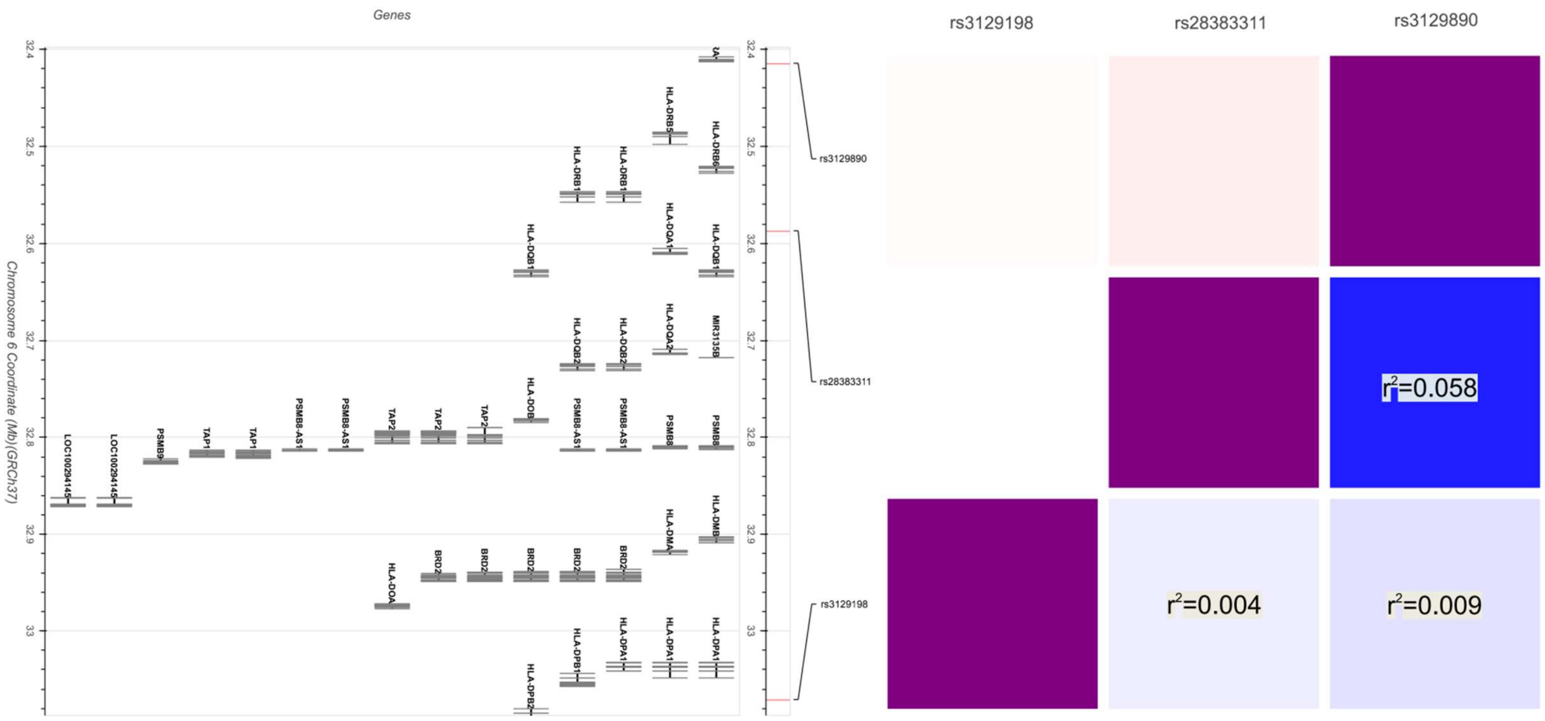

© 2022 Chen C et al. *JAMA Network Open.*

eTable 1. Meta-GWAS Results for Previously Published HL-Associated SNPs in HLA Region

SNP	Position†	Neighboring genes	Risk allele	OR	95% CI	*P*	P_{het}	I^2, %	Published Study PMID‡
rs204999	chr6:32142202:A:G	*PRRT1*	A	1.46	(1.31, 1.63)	1.43E-11	6.73E-01	0.00	22086417
rs1633096	chr6:29739490:G:T	*HLA-F*	T	1	(0.9, 1.11)	9.99E-01	2.04E-01	37.05	29196614
rs2248462	chr6:31479019:G:A	*MICB*	G	1.3	(1.16, 1.46)	6.22E-06	4.16E-01	0.00	22286212
rs2281389	chr6:33092019:A:G	*HLA-DPB1*	G	1.53	(1.38, 1.71)	8.33E-15	2.97E-01	17.67	21596858
rs2395185	chr6:32465390:G:T	*HLA-DRA*	G	1.11	(1.01, 1.22)	3.74E-02	6.73E-01	0.00	22286212
rs2734986	chr6:29850791:T:C	*HLA-A*	T	1	(0.89, 1.13)	9.37E-01	3.70E-01	0.00	22286212
rs2858870	chr6:132604474:A:G	*HLA-DRA*	A	1.11	(1.01, 1.22)	2.41E-02	8.76E-01		22086417
rs6457715	chr6:33115411:A:G	*HLA–DPB1*	A	1.04	(0.93, 1.15)	4.91E-01	7.41E-01	0.00	26404960
rs6903608	chr6:32460508:C:T	*HLA-DRA*	C	1.44	(1.31, 1.58)	1.00E-14	5.26E-01	0.00	21037568, 21596858, 22086417, 22286212, 24149102
rs6904029	chr6:29975290:G:A	*HcG9/HLA-A*	A	1.05	(0.95, 1.15)	3.72E-01	1.04E-02	78.10	22286212
rs9268528	chr6:32415331:A:G	*HLA-DRA*	G	1.37	(1.25, 1.5)	1.19E-11	1.97E-01	38.44	22086417
rs9268542	chr6:32416944:A:G	*HLA-DRA*	G	1.37	(1.25, 1.49)	1.34E-11	1.64E-01	44.65	22086417
rs9269081	chr6:32473323:A:C	*HLA-DRA*	A	1.53	(1.4, 1.69)	2.41E-19	2.90E-01	19.33	29196614
rs13196329	chr6:32357594:A:C	*C6orf10*	C	1.47	(1.07, 2.01)	1.59E-02	5.73E-02	65.02	29196614

Abbreviations: GWAS, genome-wide association study; HL, Hodgkin Lymphoma; OR, odds ratio; CI, confidence interval.
†Position according to human reference GRCh38.
Heterogeneity between data sets was assessed by *P*-value (P_{het}) and inverse variance (I^2) using the Cochran's Q statistic.
‡PMID for studies previously published the HL-associated SNP in HLA region.

© 2022 Chen C et al. *JAMA Network Open.*

eTable 2. Significant Amino Acid Changes Associated With HL in HLA Genes

Marker ID	Position[†]	Effect allele	Non effect allele	Imputed Z[‡]	r^2 pred[*]	Imputed *P*
AA_DQB1_-5_32742280_L	32634302	P	A	8.64	0.90	5.65E-18
AA_DQB1_87_32740576_LY	32632598	A	P	8.21	0.88	2.30E-16
AA_DQB1_87_32740576_F	32632598	P	A	8.11	0.89	5.23E-16
AA_DQB1_38_32740723	32632745	V	A	-7.84	0.92	4.39E-15
AA_DQB1_71_32740624_KA	32632646	P	A	-7.77	0.91	7.78E-15
AA_DQB1_57_32740666_D	32632688	P	A	7.76	0.88	8.62E-15
AA_DQB1_77_32740606	32632628	R	T	-7.69	0.91	1.48E-14
AA_DQB1_75_32740612	32632634	V	L	-7.48	0.90	7.45E-14
AA_DQB1_74_32740615_E	32632637	A	P	-7.48	0.90	7.45E-14
AA_DQB1_71_32740624_T	32632646	A	P	-7.48	0.90	7.45E-14
AA_DRB1_30_32660058_YHR	32552080	A	P	-7.28	0.94	3.22E-13
AA_DRB1_11_32660115_SPV	32552137	A	P	-7.28	0.94	3.22E-13
AA_DRB1_30_32660058_YR	32552080	A	P	-6.98	0.89	2.91E-12
AA_DRB1_30_32660058_YH	32552080	A	P	-6.96	0.94	3.30E-12
AA_DRB1_13_32660109_YF	32552131	P	A	-6.96	0.94	3.30E-12
AA_DQB1_57_32740666_AD	32632688	A	P	-6.80	0.92	1.06E-11
AA_DQB1_87_32740576_Y	32632598	P	A	-6.78	0.92	1.18E-11
AA_DQB1_57_32740666_AV	32632688	A	P	6.77	0.92	1.31E-11
AA_DQB1_87_32740576_LF	32632598	A	P	-6.70	0.92	2.02E-11
AA_DRB1_30_32660058_Y	32552080	A	P	-6.69	0.90	2.24E-11
AA_DRB1_13_32660109_SRH	32552131	A	P	-6.68	0.88	2.47E-11
AA_DRB1_30_32660058_LC	32552080	P	A	-6.55	0.94	5.81E-11
AA_DRB1_11_32660115_GL	32552137	P	A	-6.55	0.94	5.81E-11
AA_DQB1_-5_32742280_PL	32634302	A	P	-6.47	0.91	1.00E-10
AA_DQB1_-9_32742292_M	32634314	A	P	-6.46	0.91	1.05E-10
AA_DQB1_-27_32742346_A	32634368	A	P	-6.46	0.91	1.05E-10
AA_DQB1_-5_32742280_PS	32634302	A	P	6.44	0.92	1.18E-10
AA_DRB1_28_32660064_D	32552086	A	P	-6.39	0.90	1.70E-10
AA_DQB1_30_32740747_Y	32632769	P	A	6.34	0.91	2.31E-10
AA_DQB1_57_32740666_V	32632688	P	A	-6.29	0.95	3.26E-10
AA_DRB1_30_32660058_YGR	32552080	A	P	-6.23	0.90	4.59E-10
AA_DRB1_30_32660058_YHG	32552080	A	P	-6.21	0.95	5.21E-10
AA_DQB1_-9_32742292_I	32634314	P	A	-6.05	0.93	1.48E-09
AA_DQB1_224_32737107_R	32629129	P	A	-6.00	0.93	1.98E-09
AA_DQB1_125_32737913_S	32629935	P	A	-6.00	0.93	1.98E-09
AA_DQB1_116_32737940_I	32629962	P	A	-6.00	0.93	1.98E-09
AA_DQB1_71_32740624_A	32632646	P	A	-6.00	0.93	1.98E-09
AA_DQB1_-5_32742280_S	32634302	P	A	-6.00	0.93	1.98E-09

© 2022 Chen C et al. *JAMA Network Open.*

AA_DQB1_-27_32742346_S	32634368	P	A	-6.00	0.93	1.98E-09
AA_DRB1_30_32660058_YG	32552080	A	P	-5.93	0.90	3.04E-09
AA_DQB1_14_32740795_L	32632817	P	A	-5.91	0.92	3.33E-09
AA_DQB1_45_32740702	32632724	E	G	5.91	0.89	3.35E-09
AA_DQB1_14_32740795_M	32632817	A	P	-5.87	0.92	4.41E-09
AA_DQB1_116_32737940_V	32629962	A	P	-5.86	0.92	4.63E-09
AA_DQB1_125_32737913_AG	32629935	A	P	-5.85	0.92	4.85E-09
AA_DQB1_125_32737913_AS	32629935	A	P	5.80	0.92	6.73E-09
AA_DQB1_224_32737107_Q	32629129	A	P	-5.78	0.91	7.56E-09
AA_DQB1_125_32737913_G	32629935	P	A	5.69	0.92	1.29E-08
AA_DRB1_28_32660064_E	32552086	P	A	-5.61	0.91	1.97E-08
AA_DRB1_78_32659914	32551936	V	Y	-5.54	0.91	3.06E-08
AA_DRB1_30_32660058_LG	32552080	P	A	-5.54	0.91	3.06E-08
AA_DRB1_11_32660115_GD	32552137	P	A	-5.54	0.91	3.06E-08
AA_DRB1_4_32665400_Q	32557422	P	A	-5.54	0.91	3.06E-08
AA_DQB1_167_32737787_R	32629809	A	P	5.48	0.87	4.21E-08
AA_DRB1_58_32659974_A	32551996	A	P	5.46	0.82	4.73E-08

Abbreviations: HL, Hodgkin Lymphoma.

† Position refers to the genomic coordinates based on GRCh37.

‡ Z score represents HL association.

* r^2pred represents assessment of the imputation reliability at each HLA variant.

© 2022 Chen C et al. *JAMA Network Open.*

eTable 3. Previously Reported HLA Alleles Associated With HL Susceptibility

Marker ID	Position[†]	Effect allele	Non effect allele	Imputed Z[$]	r^2 pred[*]	Imputed *P*	Published Study PMID[‡]
HLA_A_01	29911991	Presence	Absence	0.14	0.97	8.88E-01	34830986
HLA_B_37	31323293	Presence	Absence	1.76	0.75	7.77E-02	22808081
HLA_DPB1_0301	33049368	Presence	Absence	2.72	0.88	6.61E-03	29196614
HLA_DQA1_0201	32608306	Presence	Absence	-4.83	0.92	1.35E-06	21596858
HLA_DQB1_0303	32631061	Presence	Absence	-3.99	0.82	6.51E-05	21596858
HLA_DQB1_0602	32631061	Presence	Absence	6.08	0.88	1.17E-09	21596858
HLA_DRB1_0701	32552064	Presence	Absence	-4.66	0.92	3.17E-06	21596858
HLA_DRB1_1501	32552064	Presence	Absence	5.84	0.89	5.28E-09	21596858

Abbreviations: HL, Hodgkin Lymphoma.

[†] Position refers to the genomic coordinates based on GRCh37.

[$] Z score represents HL association.

[*] r^2pred represents assessment of the imputation reliability at each HLA variant.

[‡] PMID for studies previously published the HL-associated HLA alleles.

© 2022 Chen C et al. *JAMA Network Open.*

eTable 4. Meta-GWAS Results for Previously Published HL-Associated SNPs in Non-HLA Region

SNP	Position†	Neighboring genes	Risk allele	OR	95% CI	*P*	P_{het}	I^2, %	Published Study PMID‡
rs1432295	chr2:60839531:G:A	*REL*	G	1.20	(1.10, 1.32)	6.42E-05	4.36E-01	0.00	21037568
rs2019960	chr8:128180025:T:C	*PVT1*	C	1.30	(1.17, 1.44)	7.05E-07	8.03E-01	0.00	21037568, 24920014
rs20541	chr5:132660272:A:G	*IL13*	A	1.19	(1.07, 1.32)	1.52E-03	6.92E-01	0.00	22286212
rs3806624	chr3:27723132:A:G	*EOMES*	G	1.21	(1.11, 1.32)	3.21E-05	7.56E-01	0.00	24149102
rs7745098	chr6:135093866:C:T	*HBS1L, MYB*	C	1.05	(0.96, 1.15)	2.78E-01	2.41E-01	29.78	24149102
rs2069757	chr5:132662721:G:A	*IL13*	A	1.19	(1.03, 1.39)	2.20E-02	3.36E-01	8.30	24920014
rs1860661	chr19:1650135:A:G	*TCF3*	NA	NA	NA	NA	NA	NA	24920014
rs4459895	chr3:188236626:A:C	*LPP*	A	1.22	(1.09, 1.37)	4.89E-04	4.02E-01	0.00	29196614
rs9482849	chr6:127967391:T:C	*PTPRK*	C	1.21	(1.07, 1.36)	1.80E-03	8.38E-01	0.00	29196614
rs6928977	chr6:135305210:T:G	*AHI1*	G	1.18	(1.07, 1.29)	4.89E-04	2.78E-01	21.81	29196614
rs3781093	chr10:8059964:T:C	*GATA3*	T	1.44	(1.26, 1.65)	1.01E-07	1.75E-01	42.67	29196614
rs112998813	chr13:114294254:T:C	*UPF3A*	T	1.12	(0.94, 1.34)	1.97E-01	7.84E-01	0.00	29196614
rs34972832	chr16:11105081:G:A	*CLEC16A*	A	1.21	(1.09, 1.35)	3.44E-04	9.24E-01	0.00	29196614
rs649775	chr6:33716536:A:G	*ITPR3, UQCC2, IP6K3*	A	1.30	(1.12, 1.51)	5.21E-04	4.83E-01	0.00	30194254
rs1002658	chr6:137660447:C:T	*OLIG3, TNFAIP3*	T	1.13	(1.01, 1.26)	3.37E-02	2.54E-01	26.97	30194254
rs7111520	chr11:111378886:G:A	*POU2AF1*	A	1.04	(0.95, 1.15)	3.81E-01	6.11E-01	0.00	30194254
rs6565176	chr16:30163605:T:C	*MAPK3, CORO1A*	T	1.08	(0.99, 1.18)	9.10E-02	8.39E-01	0.00	30194254
rs2425752	chr20:46073481:T:C	*NCOA5, CD40*	T	1.09	(0.98, 1.21)	1.21E-01	8.81E-01	0.00	30194254

Abbreviations: GWAS, genome-wide association study; HL, Hodgkin Lymphoma; OR, odds ratio; CI, confidence interval.
†Position according to human reference GRCh38.
Heterogeneity between data sets was assessed by *P*-value (P_{het}) and inverse variance (I^2) using the Cochran's Q statistic.
‡PMID for studies previously published the HL-associated SNP in non-HLA region.

© 2022 Chen C et al. *JAMA Network Open.*

eFigure 4. SNV rs117953624 near *PDGFD*

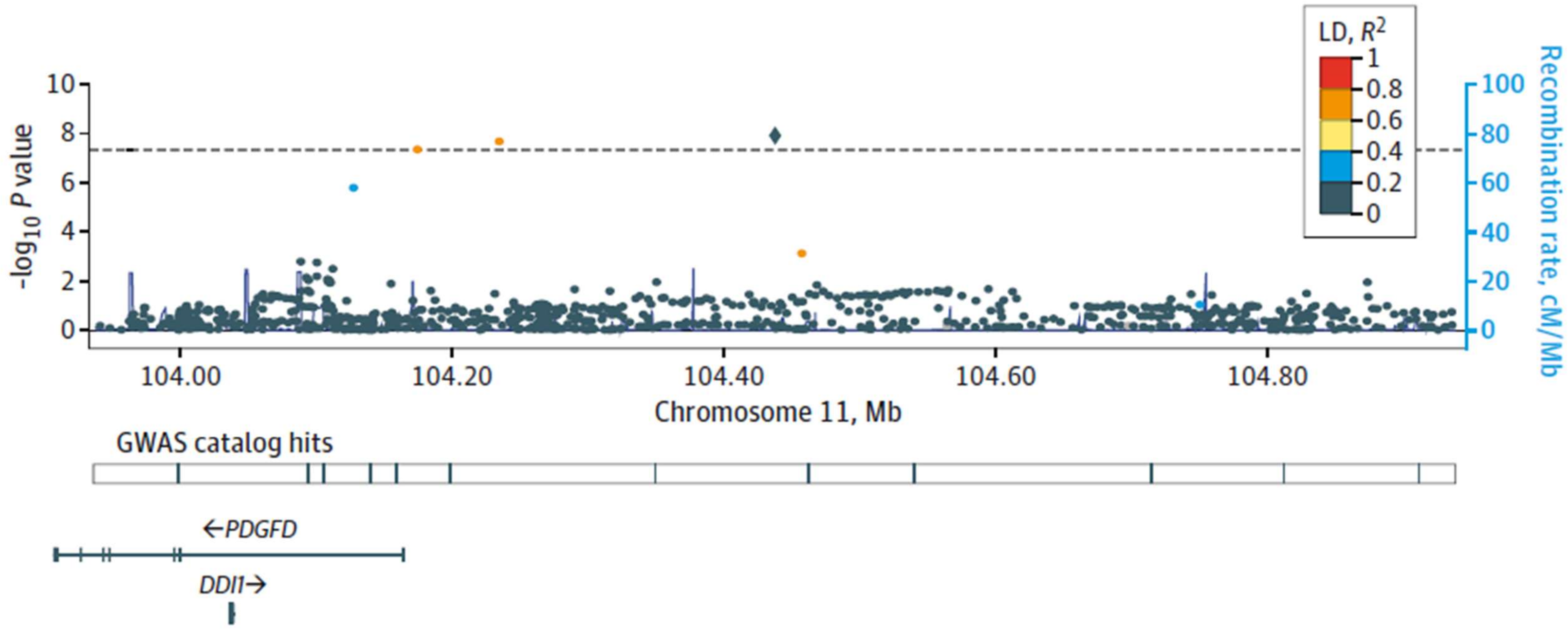

© 2022 Chen C et al. *JAMA Network Open.*
